# Draft Genome Sequences of Five *Yersinia pseudotuberculosis* ST19 Isolates and One Isolate Variant

**DOI:** 10.1128/genomeA.00122-13

**Published:** 2013-04-11

**Authors:** Mikhail E. Platonov, Yann Blouin, Vera V. Evseeva, Maxim V. Afanas’ev, Christine Pourcel, Sergey V. Balakhonov, Gilles Vergnaud, Andrey P. Anisimov

**Affiliations:** State Research Center for Applied Microbiology and Biotechnology, Obolensk, Russiaa;; Université Paris-Sud, Institut de Génétique et Microbiologie, Orsay, Franceb;; CNRS, Centre National de la Recherche Scientifique, Orsay, Francec;; Irkutsk Antiplague Research Institute of Siberia and the Far East, Irkutsk, Russiad;; DGA/MRIS—Mission pour la Recherche et l’Innovation Scientifique, Bagneux, Francee

## Abstract

We report the first draft genome sequences of five *Yersinia pseudotuberculosis* isolates of sequence type (ST) 19 and of a variant from one of the five isolates. The total length of assemblies ranged from 4,226,485 bp to 4,274,148 bp, including between 3,808 and 3,843 predicted coding sequences.

## GENOME ANNOUNCEMENT

It has been proposed that *Yersinia pestis*, the etiologic agent of plague, emerged from a *Yersinia pseudotuberculosis* O:1b ancestor (1, 2) somewhere in Eurasia (3–5). Compared to the information available about the genetically and phenotypically monomorphic *Y. pestis*, which has been the focus of detailed investigations in terms of genetic diversity, population genetics, and microevolution (3–7), relatively little is known about *Y. pseudotuberculosis* (8). This pair, *Y. pseudotuberculosis* and *Y. pestis*, represent a very promising model for the investigation of microevolution and speciation in bacteria.

To gain insights into the evolutionary origin of *Y. pestis*, we have analyzed by multilocus variable-number tandem-repeat (VNTR) analysis (MLVA) a collection of 143 *Y. pseudotuberculosis* isolates. The isolates were sampled from 1940 to 2008 from different sources in Siberia, the Far East, central Asia, and the Caucasus and northwest regions of the Russian Federation. The MLVA assay used 14 loci (ms01, ms04, ms05, ms24, ms25, ms27, ms31, ms35, ms38, ms40, ms41, ms56, ms68, and ms74) (9). The five *Y. pseudotuberculosis* isolates that clustered closest to *Y. pestis* were further analyzed by multilocus sequence typing and were shown to belong to the globally spread *Y. pseudotuberculosis* clone of sequence type 19 (ST19) O:3 (8, 10). They were isolated from small wild rodents, and interestingly, three of the selected strains were misidentified as *Y. pestis* at the moment of isolation.

Whole-genome sequencing was performed using Illumina GA IIx (Illumina, Inc.) by generating paired-end libraries with an insert size of 300 bp, according to the manufacturer’s instructions. The read length was 75 bp, and the number of reads that passed Illumina quality filters varied from 7.7 to 16 million, corresponding to 760 Mb to 1.6 Gb of high-quality data. For each genome, six million reads were *de novo* assembled using Velvet with a *k* value of 29, and the coding sequences (CDSs) were predicted using BioNumerics v6.6 (Applied Maths, Belgium). A comparison with the complete genomes of *Y. pestis* CO92 and *Y. pseudotuberculosis* strains IP31758, IP32953, and YPIII for function annotations was also performed using BioNumerics v6.6. Finally, we obtained 221 to 262 contigs for each genome. The mean total length of contigs for each strain was 4,261,235 bp, comprising, on average, 3,831 predicted CDSs. This corresponds to 90% (total length) and 93% (predicted CDSs) of the values for the complete genomes of *Y. pseudotuberculosis* strains IP31758, IP32953, and YPIII (accession no. NC_009708.1, NC_006155.1, and NC_010465.1, respectively). The average G+C content of the draft genomes is 47.53%.

The comparative genomic analysis among strains B-6796 (C-2), B-6862 (797), B-6863 (1384), B-6864 (8421-S), B-6865 (2430-1), and B-6866 (2430-2) and other available *Y. pseudotuberculosis* and *Y. pestis* isolates (i) will provide information on the speciation and evolution of *Y. pestis* and (ii) might explain the means and mechanisms of the global spread of *Y. pseudotuberculosis* clone ST19 O:3. A detailed report of a full comparative genomic analysis will be included in a future publication.

### Nucleotide sequence accession numbers. 

The draft genome sequences for the strains B-6796, B-6862, B-6863, B-6864, B-6865, and B-6866 have been included in the European Nucleotide Archive at EMBL-EBI (http://www.ebi.ac.uk/ena/data/view/) under accession no. CAQT01000001 to CAQT01000262, CAQU01000001 to CAQU01000247, CAQV01000001 to CAQV01000221, CAQW01000001 to CAQW01000237, CAQX01000001 to CAQX01000240, and CAQY01000001 to CAQY01000243, respectively.
